# Engineering β-cyclodextrin gels with nanoparticles: tunable assembly and multifunctional applications

**DOI:** 10.1039/d5na01177a

**Published:** 2026-04-30

**Authors:** Sagar Kumar Pathak, Aiswarya Sukumaran, Ioanna Chazapi, Claire Hotton, Erwan Paineau, Ravi Kumar Pujala

**Affiliations:** a Soft and Active Matter Group, Department of Physics, Indian Institute of Science Education and Research (IISER) Tirupati Andhra Pradesh 517619 India pujalaravikumar@labs.iisertirupati.ac.in; b Université Paris-Saclay, CNRS, Laboratoire de Physique des Solides Orsay 91405 France; c Université Paris-Saclay, CNRS, CEA, LLB 91191 Gif-sur-Yvette France

## Abstract

Hierarchical gels were developed through the controlled interaction of β-cyclodextrin in good–poor solvent systems, incorporating small amounts of various nanoparticles and nanoclays. These new hierarchical microstructures form through the side-by-side aggregation of β-cyclodextrin lamellar plates. They are stabilized by non-covalent interactions and facilitated by negatively charged nanoparticles or nanoclays. A systematic variation of nanoparticle concentration and solvent composition revealed that gelation occurs even at low concentrations of nanoparticles or nanoclays, significantly altering the typical phase behavior of β-cyclodextrin in DMF–water mixtures. Interestingly, a variety of differently shaped, negatively charged nanoparticles—including nanorods, nanodisks, and nanoplatelets—supported similar hierarchical self-assembly. The smart gels exhibit responsiveness to both temperature and salt, effectively removing cationic dyes. Specifically, temperature-induced phase transitions were demonstrated using three different types of nanoparticles, highlighting their potential use as temperature sensors. By combining β-cyclodextrin with nanoparticles such as cellulose nanocrystals, montmorillonite, and LAPONITE®, we developed composite gels that show improved selectivity and sensitivity for cationic dye detection.

## Abstract 2

Hierarchical supramolecular gels with tunable assembly for multifunctional applications are developed by controlling the interaction of β-cyclodextrin with small amounts of nanoparticles of various shapes.

## Graphical abstract

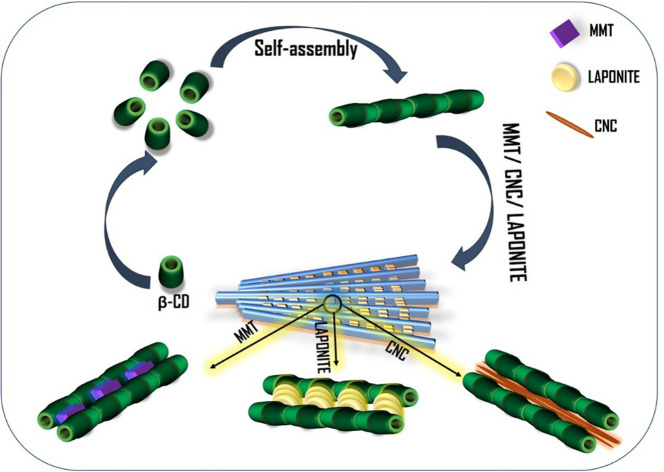

## 1. Introduction

Hierarchical organization in soft matter systems provides a versatile pathway for engineering functional materials with tunable architectures and emergent properties.^1^ By integrating order across multiple length scales through the spontaneous self-assembly of amphiphilic molecules,^2^ these systems bridge molecular design with macroscopic performance, enabling applications in sensing, environmental remediation, and stimuli-responsive technologies.^3–7^ These structures, classified as a specific type of soft material, can be constructed utilizing gelators such as polymers, natural polysaccharides, low-molecular-weight organic compounds, and other similar materials.^8,9^ Among the various gelators, β-cyclodextrin (β-CD) emerges as a particularly promising candidate for gel formation. β-CD is a cyclic oligosaccharide comprising seven glucopyranose units, characterized by its hydrophobic cavity and hydrophilic exterior.^10–12^ Due to its distinctive structure, β-CD is capable of forming inclusion complexes with appropriate guest molecules through host–guest interactions, thereby serving as an outstanding gelator for the creation of functional gels.^13–16^ Owing to its hydrophilic outer surface and end faces, β-CD readily forms hydrogen-bonded channel-type stacks in the presence of water. Screening of various solvents identified *N*,*N*-dimethylformamide (DMF), dimethyl sulfoxide, and *N*,*N*-dimethylacetamide as effective media for gel formation.^17^ β-CD dissolves well in these solvents, and subsequent addition of water at room temperature induces stable gelation *via* channel-type stacking into cage-like assembly. This process is characterized by water, acting as a poor solvent, which prompts the self-assembly of β-CD into a stable gel. This unique characteristic of β-CD offers promising prospects for the development of advanced materials applicable in drug delivery, environmental remediation, and stimuli-responsive systems.^18–20^

Stimuli-responsive supramolecular assembly driven by noncovalent interactions has garnered increasing interest for fabricating and controlling hierarchical micro- and nanostructures due to its simplicity and versatility. β-Cyclodextrin (β-CD) exhibits rich morphological diversity, including lamellar plates formed *via* side-by-side alignment stabilized by intermolecular hydrogen bonding^21^ and tubular aggregates generated through head-to-tail stacking combined with hydrophobic guest inclusion.^22^ Previous studies have demonstrated the incorporation of metal ions and graphene oxide into β-CD assemblies to modulate their mechanical properties and stimuli-responsiveness.^23,24^ However, such systems rely on specific nanomaterials and remain inherently system-dependent, limiting broader control over β-CD self-assembly. In contrast, the present work introduces a generalizable strategy in which structurally diverse, negatively charged nanoparticles—including nanoclays and nanocellulose—direct β-CD assembly within a controlled good–poor solvent framework. This enables systematic tuning of gelation behavior, novel feather mimicking hierarchical architecture, and multi-stimuli responsiveness, thereby advancing beyond system-specific approaches and providing a versatile platform for designing functional supramolecular gels.^25–28^

**Fig. 1:**
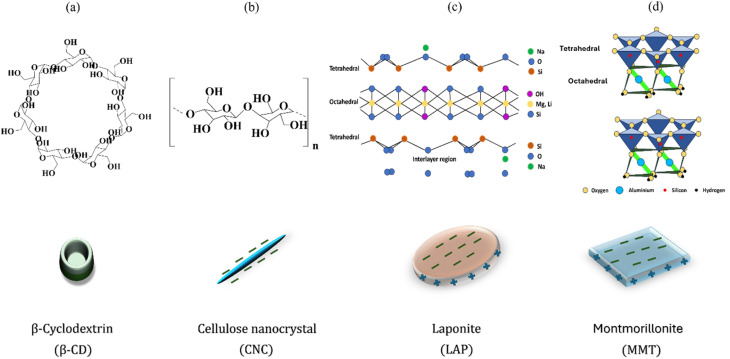
Structural representations of (a) β-cyclodextrin, (b) cellulose nanocrystals, (c) LAPONITE®, and (d) montmorillonite, highlighting their molecular frameworks and characteristic morphologies used in hierarchical gel assembly.

We selected negatively charged nanoparticles of various shapes and sizes to investigate their impact on the self-assembly of β-cyclodextrin (β-CD). The nanoparticles we examined include cellulose nanocrystals (CNC) and two types of smectite clay minerals: montmorillonite (MMT) and LAPONITE® (LAP). Cellulose is a linear polysaccharide composed of repeating d-glucopyranose units linked by 1,4-β-glycosidic bonds, as illustrated in Fig. 1.^29,30^ It forms rigid, rod-like particles with needle-like structures, which contain monocrystalline domains extending up to 200–400 nm in length.^31^ In contrast, LAPONITE® and montmorillonite have a sandwich-like structure consisting of an octahedral sheet flanked by two tetrahedral silica sheets.^32–34^ These clays are composed of plate-shaped nanoparticles that are 1 nm thick and possess a large, permanently charged face due to isomorphic substitution, primarily occurring in the octahedral sheet (Fig. 1). Typically, LAPONITE® has a hydrodynamic diameter of 50–70 nm, while MMT has a diameter ranging from 250–350 nm (Fig. S1).

In this study, nanoparticles such as CNC, MMT, and LAP were utilized to create smart, responsive hybrid gel systems, using β-cyclodextrin (β-CD) as the gelator. Uniform microstructures were observed to begin forming with short tips composed of β-CD channel-like assemblies. These nanoparticles were integrated with β-CD plates to create a spherulite-like hierarchical microstructure. The impact of varying DMF–water solvent ratios on the phase behavior of the system was carefully investigated. Rheological studies were conducted to evaluate the gel strength and viscoelastic properties. The gel systems containing different negatively charged nanoparticles demonstrated responsiveness to salt and temperature, highlighting their significance for sensing applications. β-CD/nanomaterial composites exhibited enhanced selectivity and sensitivity in detecting cationic dyes due to the synergistic combination of host–guest chemistry and the high surface area of the nanostructures. These attributes present a promising platform for the development of advanced environmental monitoring and sensing technologies.

## 2. Experimental methods

### 2.1 Sample preparation

β-Cyclodextrin (β-CD, C_42_H_70_O_35_) and *N*,*N*-dimethylformamide (DMF) were purchased from Sigma-Aldrich (India) and used without further modification. Cellulose nanocrystal (CNC) powder was obtained from Celluforce (Canada), sodium montmorillonite (Na^+^–MMT) from Southern Clay Products (USA), and LAPONITE® powder from BYK Additives (Germany). All materials were used as received. CNC particles exhibited sizes of 200–400 nm with a zeta potential of −31.6 mV. MMT particles ranged from 250–350 nm with a zeta potential of −37.5 mV, while LAP showed a hydrodynamic diameter of 50–70 nm and a zeta potential of −20 mV. Milli-Q (MQ) water was used to prepare CNC, MMT, and LAP suspensions (0.5–1.5 wt%) under magnetic stirring to ensure uniform dispersion (Fig. S1).

Composite samples were prepared using the good/poor solvent method as proposed by Ma M. *et al.*^23^ Typically, a stock solution of 30% (w/v) β-CD in DMF was prepared by continuous stirring and ultrasonic treatment, resulting in a transparent dispersion. As water is a poor solvent for β-CD, when an equal volume of nanoparticles (CNC, MMT, or LAP) in MQ-water was added to the stock solution, the sample turned into a white gel within 5 minutes for CNC and LAP, and a pale-yellow gel for MMT.

### 2.2 Sample characterization

Solid-state characterization of pure β-CD gels, pristine nanoparticles, and their composite systems was carried out using powder X-ray diffraction (XRD). Crystal structures were analyzed using a Rigaku SmartLab diffractometer (Cu Kβ-filter, 42 kV, 120 mA) over a 2*θ* range of 5–60° with a scan rate of 2° min^−1^. FTIR spectra were recorded on a Bruker ALPHA spectrometer in transmission mode using KBr pellets, over the range of 4000–450 cm^−1^ at a resolution of 2 cm^−1^, with 50 scans averaged. UV-Vis absorption spectra for dye sensing were collected using an Agilent Cary Series spectrophotometer with a 3 mL quartz cuvette, in the wavelength range of 450–800 nm, at various time intervals.

### 2.3 Microstructural properties of the gels

Polarized optical microscopy (POM) was conducted using a polarizing microscope (ECLIPSE LV100N POL, Nikon). Fluorescence imaging was performed on a Nikon Eclipse Ti2 inverted fluorescence microscope using a Cy5 filter set for methylene blue and a FITC filter set for Basic Orange 14 dye. Scanning electron microscopy (SEM) images were obtained with a Zeiss Supra55VP, employing an acceleration voltage of 2 keV. The samples were placed on a UV-treated silicon wafer and coated with a 15 nm layer of gold, followed by a 5 nm layer of titanium to prevent charging effects. Small-angle X-ray scattering (SAXS) experiments were performed at the SWING beamline of the SOLEIL synchrotron in Orsay, France, using a fixed energy of 12 keV. The scattering patterns were recorded on a two-dimensional detector (Eiger 4M, Dectris Ltd, Switzerland), which was positioned approximately 6.2 m from the sample within a vacuum tunnel. For these measurements, the dispersions were transferred into borosilicate glass capillaries (WJMGlas/Müller GmbH, Germany), which were flame-sealed and stored vertically prior to the experiments.

### 2.4 Rheology

All rheological measurements were conducted using Anton Paar Modular Compact Rheometer (MCR-302). The tests utilized a cone-plate geometry (CP-25) with a diameter of 25 mm and a gap of 0.104 mm, and were performed in both rotational and oscillatory modes. For the rotational tests, flow curves were analyzed, examining shear stress and viscosity as functions of shear rate. The viscoelastic properties of the samples were further explored through detailed oscillatory tests, including amplitude sweep and frequency sweep experiments. The amplitude sweep was conducted at a constant angular frequency (*ω*) of 10 rad s^−1^, while the frequency sweep was performed at a constant shear within the linear viscoelastic region (LVR) for all samples.

## 3. Results and discussions

### 3.1 Phase behavior of β-CD in DMF and water

**Fig. 2:**
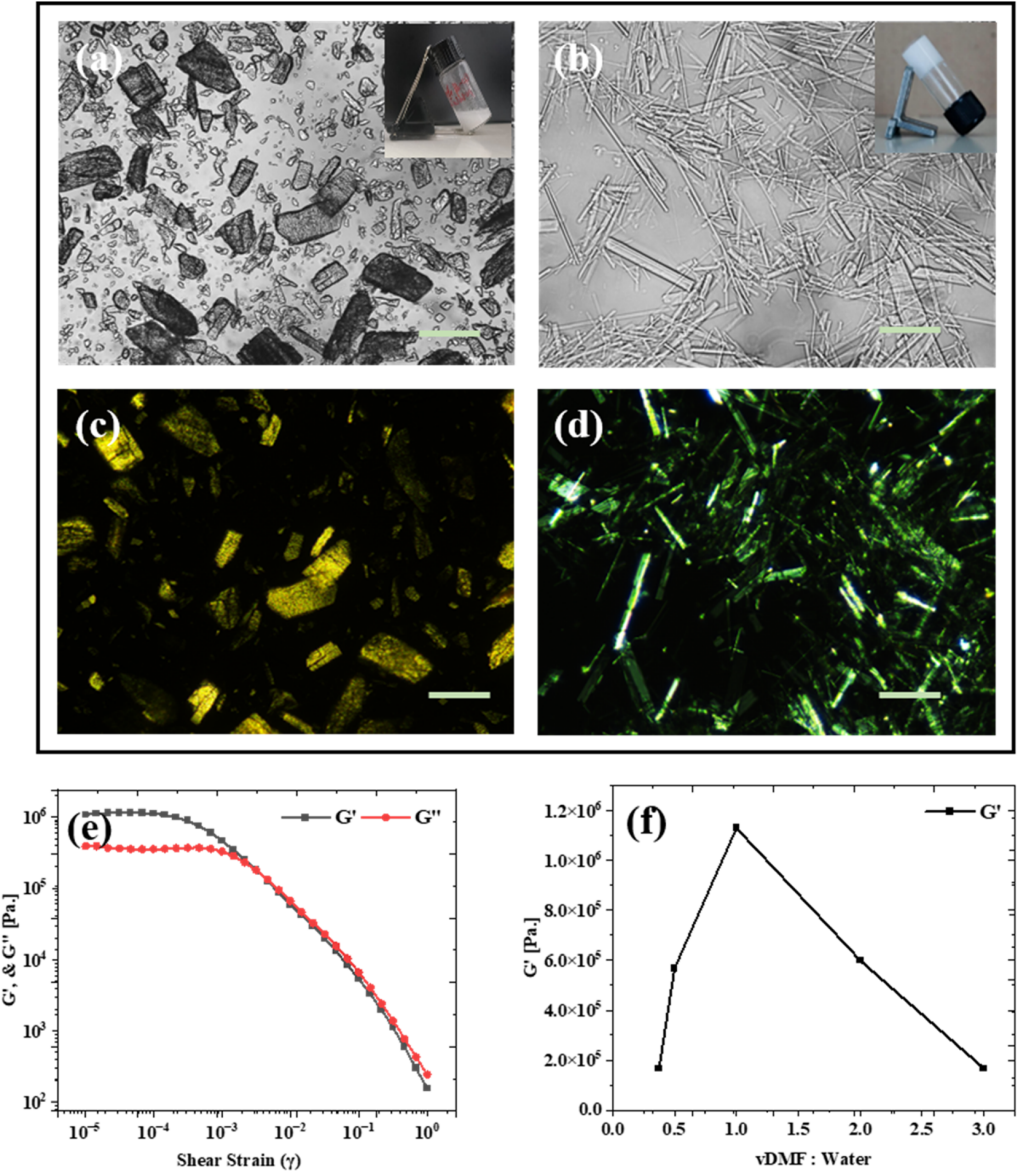
Polarized microscope image taken with bright field and crossed polarizers, respectively, (a) and (c) β-CD molecules dissolved in water (crystal phase), and (b) and (d) for β-CD in a 1 : 1 volume ratio of DMF/water mixture (gel phase), (scale bar-100 µm). (e) Rheological evidence (amplitude sweep) of gel formation in a 1 : 1 DMF and water volume ratio for constant angular frequency = 10 rad s^−1^. (f) Storage modulus (*G*′) of β-CD for different volume ratios of good–poor solvent.

In water, β-cyclodextrin (β-CD) forms plate-like, non-uniform crystalline assemblies that can vary in size from hundreds of nanometers to several hundred micrometers, as demonstrated by polarized optical microscopy (Fig. 2a–d). The formation of these gels is favored within a specific range of β-CD concentrations and solvent quality. Generally, the weight ratio of the gelator within the entire gel system should not exceed 5% to 10%, as very high concentrations of the gelator could lead to insolubility in water.^35–37^ In our study, we used a 30% β-cyclodextrin concentration, which is fully soluble in DMF (dimethylformamide), resulting in a transparent solution. Additionally, the volume ratios of DMF to water can be adjusted from 2 : 8 to 7 : 3 to facilitate the formation of a stable gel.^17^

The amplitude sweep profile (Fig. 2e) of the β-CD gel illustrates its viscoelastic characteristics, where rheology measures the balance between elastic (*G*′) and viscous (*G*″) responses under applied deformation. A linear viscoelastic region (LVER, 10^−5^–10^−2^ strain) was observed, within which *G*′ remains nearly constant at ∼1.13 × 10^6^ Pa and exceeds *G*″, signifying a predominantly elastic, solid-like network stabilized by extensive intermolecular hydrogen bonding. Beyond the critical strain (∼10^−3^), a gradual decline in both moduli indicates microstructural softening due to partial disruption of physical cross-links. As the applied strain increased beyond ∼10^−2^ (*γ*), a gradual decline in *G*′ was observed, signalling the onset of nonlinear viscoelastic behaviour. *G*″ also showed a decrease at slightly higher strains, and at approximately *γ* ≈ 0.3%, a crossover between *G*′ and *G*″ was observed. This *G*′/*G*″ crossover point is indicative of a yielding transition, where the material behaviour shifts from predominantly elastic to predominantly viscous. This transition suggests that the internal structure of the material begins to break down under increasing deformation, allowing it to flow. Beyond the crossover point (*γ* > 0.3%), both moduli decreased sharply with increasing strain, characteristic of shear-thinning or soft-solid yielding behaviour. The decline in both *G*′ and *G*″ at high strains reflects the structural breakdown and energy dissipation that occur under large deformations. Fig. 2f suggests the maximum gel strength occurred for a 1 : 1 volume ratio of β-CD (DMF) and water.

### 3.2 Development of hierarchical microstructures with β-CD and nanoparticles

**Fig. 3:**
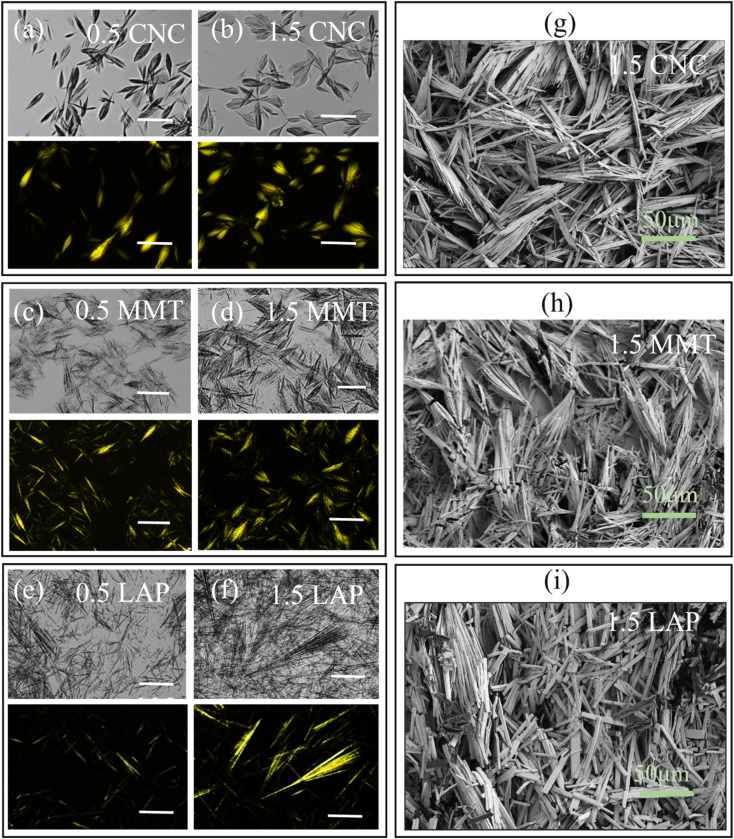
Polarized optical microscopy images of β-CD/CNC (a) and (b) and β-CD/MMT (c) and (d), and β-CD/LAP (e) and (f) composites at varying nanoparticle concentrations (0.5 wt% and 1.5 wt%), captured under bright-field (top) and cross-polarized light conditions (bottom). (g)–(i) SEM images of β-CD/CNC, β-CD/MMT, and β-CD/LAP composite systems, respectively. All scale bars represent-50 µm.

Fig. 3 illustrates the progressive changes in hierarchical microstructure as the concentrations of CNC, MMT, or LAP increase from 0.5 wt% to 1.5 wt%. At a lower CNC concentration of 0.5 wt%, a well-defined hierarchical network develops (Fig. 3a). In contrast, at a higher CNC concentration of 1.5 wt%, stronger interactions between β-CD units and CNC lead to wider and more interconnected microstructural domains (Fig. 3b). Increasing the CNC content to 1.5 wt% significantly enhances the supramolecular assembly of β-CD with CNC, expanding the hierarchical framework further. A similar hierarchical structural development is evident across MMT concentrations, ranging from 0.5 wt% to 1.5 wt% (Fig. 3c and d). Additionally, the incorporation of LAP significantly influences the self-assembly properties of the β-CD/LAP composite system. At lower LAP concentrations, the microstructure shows random, plate-like formations with weak birefringence observable under polarized optical microscopy, indicating minimal structural organization (Fig. 3e). However, as LAP content increases, birefringent textures gradually appear, signalling the formation of hierarchically structured domains driven by the interaction between LAP and β-CD channel-type microplates (Fig. 3f).^37^ Microscopic analysis revealed the formation of distinctive bow-tie-shaped β-CD microstructures arising from oriented self-assembly and directional crystallization guided by nanomaterial templates (CNC, MMT, LAP), highlighting the role of surface-induced anisotropic growth in shaping supramolecular architectures (Fig. S2). We also aim to explore the interactions among these components and the corresponding rheological behavior of the composites. SEM images in Fig. 3g–i showcase well-defined platelets formed from channel structures within the β-CD gel microstructure. The SEM images reveal that these platelets attach side by side, emphasizing their organized assembly.

### 3.3 Characterization of hierarchical gels

#### FTIR analysis

**Fig. 4:**
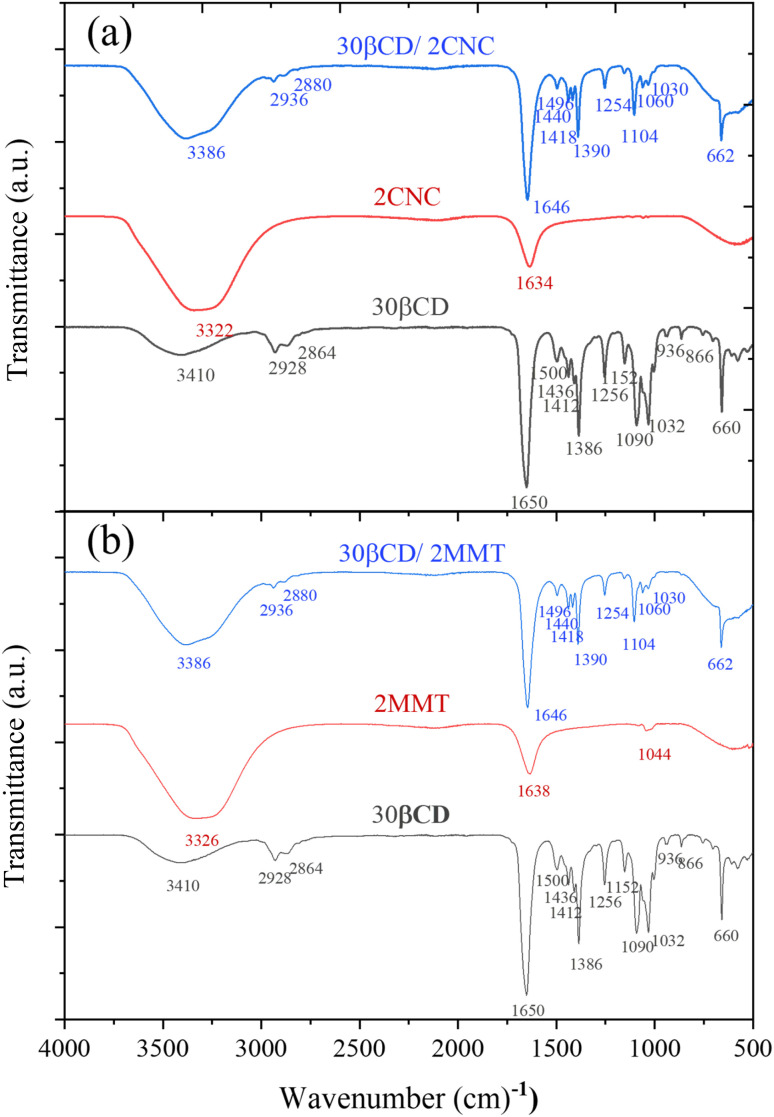
FTIR spectra of (a) β-CD/CNC, and (b) β-CD/MMT composites.

Fourier-transform infrared spectroscopy was employed to analyse the functional groups and interfacial interactions within the β-cyclodextrin (β-CD) composite systems (Fig. 4). The FTIR spectrum of pure β-CD shows a broad O–H stretching vibration in the range of 3300–3410 cm^−1^, attributed to extensive hydrogen bonding among hydroxyl groups.^38^ The band at 2920–2936 cm^−1^ corresponds to C–H stretching, while the strong peaks between 1020 and 1150 cm^−1^ were due to C–O–C and C–C stretching within the glucopyranose rings.^39^ For pure CNC, the spectrum shows an O–H stretching band near 3322 cm^−1^ and prominent C–O stretching vibrations between 1000–1160 cm^−1^, typical of cellulose. In the β-CD/CNC composite, the O–H band broadens and shifts slightly, while the C–O–C stretching region (∼1104 cm^−1^) shows minor shifts and intensity changes, indicating stronger hydrogen bonding and possible supramolecular complexation.^40^ The absence of new peaks confirms that the interaction was predominantly physical rather than covalent.

MMT displays a broad O–H stretching band near 3326 cm^−1^ from interlayer water, an H–O–H bending vibration at 1638 cm^−1^, and a strong Si–O stretching band at 1044 cm^−1^. Bands at 915 cm^−1^ and 525 cm^−1^ correspond to Al–Al–OH and Si–O–Al bending vibrations, respectively, typical for layered silicates.^41^ In the β-CD/MMT composite, the O–H stretching band shifts to 3386 cm^−1^ and the Si–O stretching band shifts to 1030 cm^−1^, with band broadening and reduced intensity, suggesting hydrogen bonding or electrostatic interactions between β-CD and MMT layers, consistent with intercalation or surface adsorption.^42,43^ The FTIR spectrum of LAPONITE® shows characteristic silicate features, including a broad O–H band near 3410 cm^−1^, an –OH deformation band at ∼1638 cm^−1^, and a strong Si–O stretching vibration at 1004 cm^−1^ (ref. 41 and 44) (Fig. S3). In the resulting β-CD/LAP composite, these bands broaden and shift slightly, indicating hydrogen bonding between β-CD hydroxyl groups and LAP silanol sites, and possible surface adsorption or partial intercalation.^45^ Thus, FTIR analysis conclusively indicates the presence of both organic and inorganic elements, highlighting hydrogen bonding as the predominant interaction mechanism responsible for the stabilisation of the composite structure.

Zeta potential analysis (Fig. S4a–c) of the β-CD/nanoparticle system shows a decrease in zeta potential with increasing nanoparticle concentration, confirming the occurrence of electrostatic interactions between β-CD and the nanoparticles.

#### Rheological measurements

**Fig. 5:**
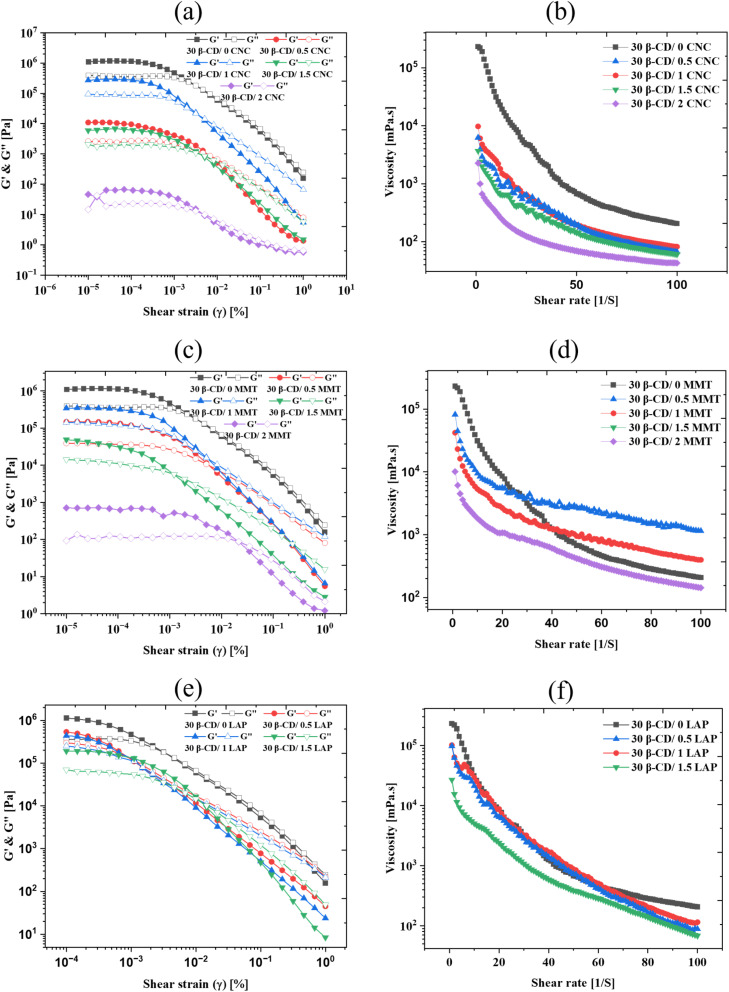
Evolution of (left) amplitude sweep (constant angular frequency = 10 rad s^−1^), and (right) flow curve of β-CD dispersions prepared with different concentrations of (a) and (b) CNC. (c) and (d) MMT, and (e) and (f) LAP respectively. Different concentrations were represented with various colours as storage modulus *G*′ (closed symbols) and loss modulus *G*″ (open symbols).

In the following, we investigated the viscoelastic characteristics and hierarchical structures of β-CD-based supramolecular gels using amplitude sweeps, frequency sweeps, and flow measurements (Fig. 5).

Increasing CNC concentration from 0.5 wt% to 2 wt% influenced the gel's mechanical robustness and internal structure (Fig. 5a and b). At a lower CNC content (0.5 wt%), the gel exhibited a well-defined linear viscoelastic region (LVR) with a *G*′ value of approximately 10^4^ Pa, reflecting a moderately elastic network. Increasing CNC to 1.5 wt% lowered *G*′ significantly (to ∼10^3^ Pa), indicating reduced network formation, by more extended hierarchical microstructures. However, at the highest CNC loading (2 wt%), *G*′ decreased to ∼10^2^ Pa, suggesting that excessive CNC may interfere with optimal crosslinking, leading to a weakened network.^46,47^ The amplitude sweeps also showed that LVR expanded with CNC loading, implying greater deformation tolerance before network breakdown. Frequency sweeps showed that *G*′′ exceeded *G*′ at higher CNC concentrations, indicating more liquid-like behaviour (Fig. S5). The flow curves again confirmed pronounced shear-thinning, consistent with the progressive disruption of the self-assembled structure under shear.^47–49^

A similar trend was observed for the β-CD/MMT system. Amplitude sweep data (Fig. 5c) revealed a high initial *G*′ (∼10^6^ Pa) at low MMT content (0.5 wt%), indicative of a strong, elastic gel network.^49,50^ However, *G*′ values declined significantly with increasing MMT concentration, dropping to ∼10^3^ Pa at 2 wt%. This reduction suggests that excessive MMT disrupted the β-CD hydrogen-bonded channels, weakening the overall supramolecular framework. Notably, all formulations exhibited a critical strain threshold (∼0.01), beyond which *G*′ declined sharply, indicating the onset of network failure and transition to a more fluid-like behavior. Flow curves (Fig. 5d) again demonstrated pronounced shear-thinning behavior, typical of physically cross-linked supramolecular gels. While all MMT-containing gels displayed non-Newtonian characteristics, the steepness of the viscosity decline varied with MMT concentration, reflecting differences in structural integrity and filler–matrix interactions.^60^

In the β-CD/LAP system, rheological analysis revealed consistent effects as LAP concentrations varied. Specifically, as the LAP content increased from 0.5 wt% to 1.5 wt%, there was a noticeable reduction in the storage modulus (*G*′) values, decreasing from approximately 10^6^ Pa to 10^4^ Pa. This reduction indicates a decrease in rigidity due to the partial disintegration of the β-CD structured network at higher filler loadings. Frequency sweeps consistently showed a characteristic of a viscoelastic liquid, where the loss modulus (*G*′′) exceeded the storage modulus (*G*′) across all frequencies (refer to Fig. S5). Additionally, flow curves (Fig. 5f) displayed pronounced shear-thinning behavior, where the viscosity remained high at lower shear rates but significantly decreased as shear rates increased. This behavior is typical of physically cross-linked gels. In comparison to the CNC and MMT systems, the changes in rheological parameters (*G*′, *G*′′, and viscosity, *η*) for the β-CD/LAP composite were less pronounced. Scanning electron microscopy (SEM) and polarized optical microscopy (POM) images (Fig. 3c, h and i) further confirmed that the development of microstructures was less prominent, resulting in only minor variations in viscoelasticity with increasing LAP concentration.

#### SAXS and XRD analysis

**Fig. 6:**
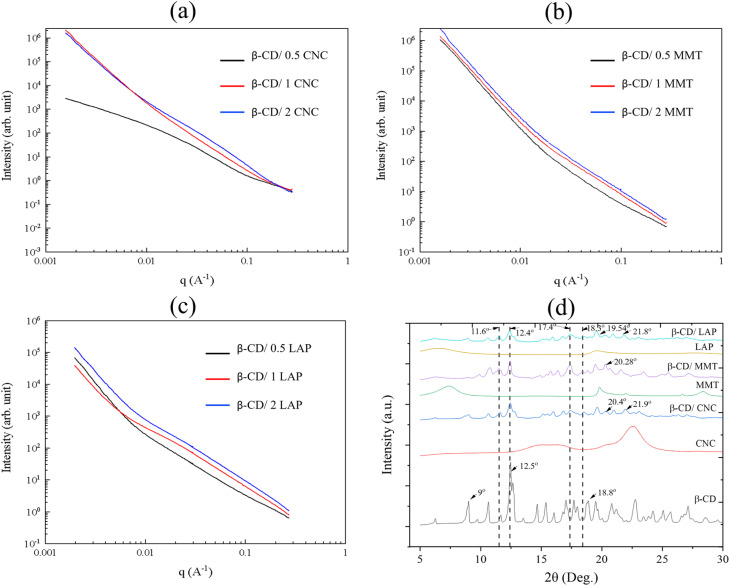
(a)–(c) SAXS profile of β-CD/CNC, β-CD/MMT, and β-CD/LAP, respectively. (d) Powder XRD patterns comparison of β-CD, CNC, β-CD/CNC, MMT, β-CD/MMT, LAP, and β-CD/LAP.

SAXS patterns of the different β-CD/nanoparticles composites were displayed in Fig. 6a–c. Except for the sample prepared at 0.5 wt% CNC, all SAXS curves follow a *Q*^−4^ power-law decay at low *Q*-values with a noticeable change in the slope for *Q* > 0.01 Å^−1^. The increase in the concentration of CNC or clay nanoparticles only affects the intensity of scattering curves, as expected. However, we did not detect any characteristic correlation distances, whatever the composition of β-CD/nanoparticles composites. X-ray diffraction (XRD) was extensively utilized as a technique in the analysis of inclusion complexes to evaluate their structural characteristics.^52^ β-CD mostly exhibits two characteristic patterns in crystal structures: cage-type and channel-type. Fig. 6d illustrates that β-CD exhibits prominent peaks at 9°, 12.9°, and 18.8°, originating from its cage-like molecular structure.^57^ Additionally, major peaks were observed at 12.4°, 17.2°, 18.3°, and 11.6°, indicating a head-to-head channel-type arrangement of β-CD molecules across the composite systems involving CNC, MMT, and LAP.^53,57–59^ Accordingly, Fig. S6 and Table S1 show the (001) plane with an enhanced *d*-spacing from 13.87 to 14.11 Å, 13.57 to 14.03 Å and 13.80 to 14.12 Å for LAP, CNC, and MMT, respectively, while increasing the ratio between β-CD and nanoparticles as a result of the intercalation of β-CD into the interlayer spacing of 2D clay nanosheets.^54^ New characterized peaks 21.9°, 21.55°, and 21° are observed for β-CD/CNC, β-CD/MMT, and β-CD/LAP composite systems, respectively, which can be due to the presence of nanoparticles in the system.

### 3.4 Phase diagrams and molecular discussion

Experimental observations have shown that gel formation in β-cyclodextrin (β-CD) systems is highly dependent on the concentration of β-CD and the ratio of good to poor solvents.^55^ Gelation occurs exclusively within a narrow range of solvent ratios, specifically at DMF/H_2_O volume ratios between 1 : 4 and 7 : 3, with a critical gelation concentration (CGC) established at 1.7 wt%. To explore the influence of nanoparticles on this gelation process, small amounts of nanoparticles were dispersed in Milli-Q water and then combined with β-CD dissolved in DMF. The effects of both solvent composition and the concentration of nanomaterials on phase behavior—including transitions between gelation and sedimentation—were systematically studied.

**Fig. 7:**
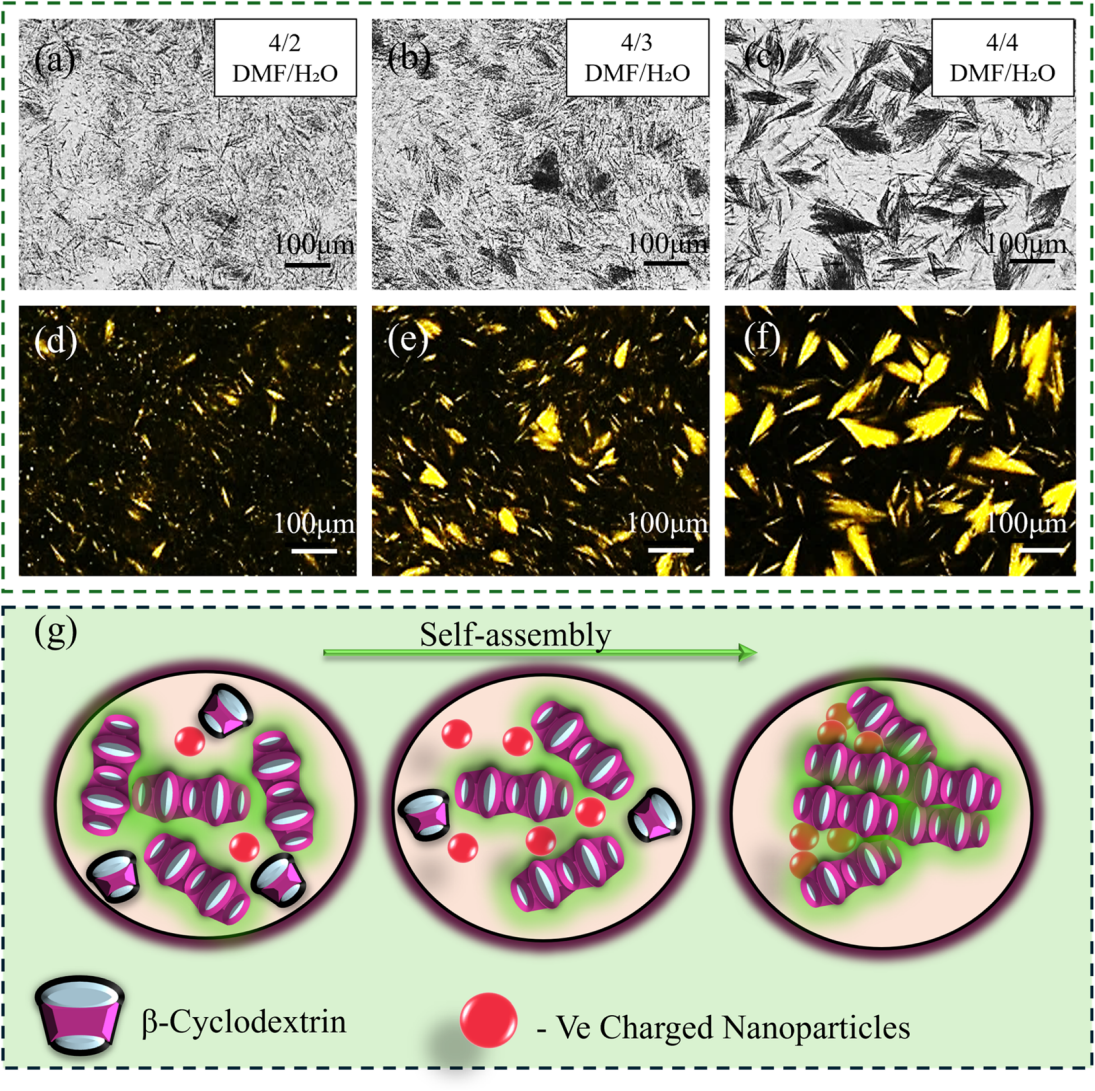
(a)–(c) Optical images and (d)–(f) polarized optical images between crossed polarizers of formation of hierarchical microstructures in 30 wt% β-CD/2 wt% MMT composite gel, with varying volume ratio of β-CD (DMF), and MMT (water) from 4 : 2 to 4 : 4. (Scale bar −100 µm); (g) schematic representation of the general gelation mechanism in the composite system, where increasing the nanoparticle (water) content within a fixed β-CD/DMF solution promotes supramolecular aggregation and network formation.

Fig. 7a–f, demonstrates the formation of a hierarchical microstructure as the volume ratio of β-CD (DMF)/nanomaterials (water) increases for a constant nanomaterial concentration (2 wt% MMT). At a low ratio (4 : 1), gelation is predominant due to the channel-like assembly of β-CD in response to the good–poor solvent interaction. As the ratio increases, the laminar channels formed by β-CD reassemble in a hierarchical manner (for 4 : 3 and 4 : 4).

**Fig. 8:**
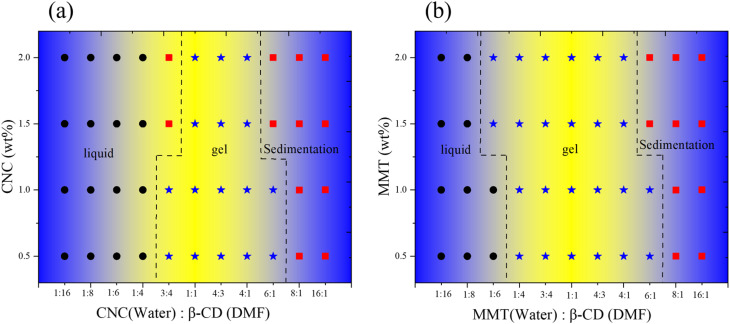
(a) and (b) Represent the general phase behavior of the β-CD/CNC and β-CD/MMT composite systems in varying concentrations of solvents.

To elucidate the role of nanoparticles in the development of hierarchical microstructures, the volume ratio of β-CD(DMF) to nanomaterial (in water) was varied from 1 : 16 to 16 : 1, and MMT concentration was varied from 0.5 to 2 wt% (Fig. 8a). At a high water content (1 : 16 volume ratio), the system remained a transparent liquid, indicating that neither gelation nor microstructure formation occurred under these conditions. However, when the volume ratio was adjusted to 1 : 6–4 : 1 with 30 wt% β-CD(DMF) and 1.5–2 wt% MMT, β-CD molecules were observed to self-assemble into channel-like structures, facilitated by the presence of MMT, leading to the formation of stable gels. At lower MMT concentrations (0.5–1 wt%), gelation was initiated at solvent ratios of 1 : 4–6 : 1, confirming the critical role of MMT in modulating the gelation threshold. The corresponding phase diagram (Fig. 8a) clearly demonstrates the shift in gelation behavior caused by the presence of MMT, relative to the pure β-CD/DMF/H_2_O system.

A similar trend was observed for the other negatively charged clay mineral. In the case of LAPONITE®, gel formation occurred between 1 : 6 and 1 : 4 volume ratios at 0.5–1.5 wt% and shifted slightly to 1 : 4–6 : 1 at 2 wt% (Fig. S7). In contrast, with CNC, gelation occurred at a 3 : 4–6 : 1 ratio for 0.5–1.5 wt% and shifted to a 1 : 1–4 : 1 ratio for 2 wt%, highlighting that the gelation window was dependent on both nanomaterial type and concentration (Fig. 8b).

The results suggest that even small volume amounts of MMT significantly promote gel formation by enabling the formation of a robust, interconnected network—something not achieved by β-CD and water alone. Furthermore, stable feather-like microstructures were observed *via* microscopic images (Fig. 7) when the solvent system approached a 1 : 1 DMF/H_2_O ratio, indicating optimized conditions for hierarchical self-assembly in the presence of nanoclays.

**Fig. 9:**
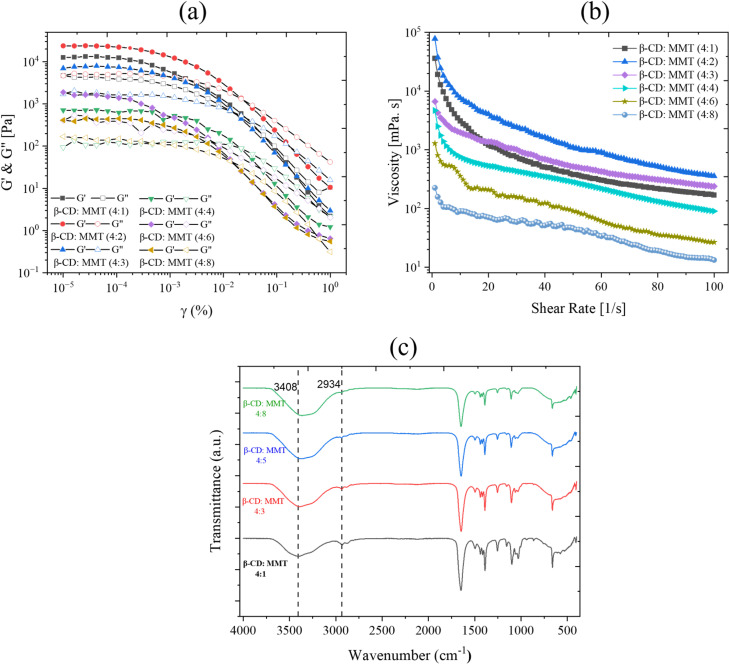
(a) Amplitude sweep, and (b) flow curve measurements of β-CD with different concentrations of volume ratio of β-CD(DMF), and MMT (water) represented with various colours as storage modulus *G*′ (closed symbols) and loss modulus *G*″ (open symbols). (c) FTIR spectra of β-CD(DMF)/MMT composite, with varying volume ratio of β-CD(DMF), and MMT (water) from 4 : 1 to 4 : 4.

The oscillatory measurements of storage modulus (*G*′) and loss modulus (*G*″) as a function of strain were conducted for constant angular frequency = 10 rad s^−1^, with varying volume ratio of β-CD(DMF), and MMT (water) from 4 : 1 to 4 : 8 for fixed 2 wt% MMT concentration (Fig. 9a). In the lower strain region, *G*′ was observed to be greater than *G*″ up to a strain of 0.01%. This indicates a gel of weak structure, primarily formed by weak interactions. Notably, the volume ratio of β-CD(DMF) and MMT (water) increases from 4 : 1 to 4 : 4; there was a significant enhancement in the elastic strength, which ranged from 10^2^ to 10^5^ Pa. Elastic strength increases from a 4 : 1 to a 4 : 2 volume ratio. 4 : 2 volume ratio of β-CD(DMF)/MMT(Water), 4 : 2 volume ratio shows the maximum elastic strength. It shows a decreasing trend in the volume ratio, from 4 : 3 to 4 : 8.

To further investigate the role of MMT and good–poor solvent interaction in conjunction with β-cyclodextrin (β-CD), we conducted flow curve measurements (Fig. 9b) by varying the shear rate at different MMT and solvent concentrations. The flow curve exhibited shear-thinning behavior at higher shear rates, indicating that the structures formed by β-CD in water realigned along the shear direction without disruption.

FTIR spectroscopy (Fig. 9c) was employed to examine hydrogen bonding in β-CD (DMF)/MMT (water) systems with varying volume ratios. A broad O–H stretching band (3200–3600 cm^−1^) shifted to lower wavenumbers with increasing MMT content, indicating stronger hydrogen bonding interactions. Apart from this shift, no major spectral changes were observed, confirming that β-CD/MMT interactions were primarily driven by hydrogen bonding.

### 3.5 Molecular discussion

**Fig. 10:**
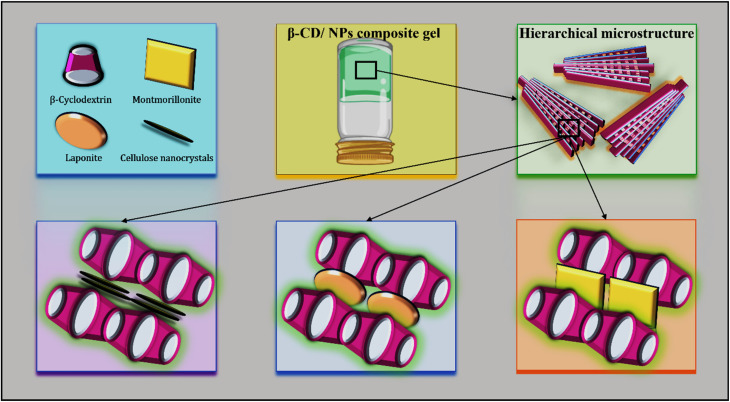
Schematic representation of the self-assembly mechanism in β-CD/nanoparticle composite gels. β-Cyclodextrin (β-CD) forms channel-type structures that act as primary building blocks. Upon incorporation of nanoparticles such as cellulose nanocrystals, montmorillonite, and LAPONITE®, interfacial interactions and hydrogen bonding promote hierarchical alignment.

The mechanism behind the formation of spherulitic microstructures in a composite system made up of β-cyclodextrin (β-CD) and nanoparticles—such as CNC, MMT, or LAP—was systematically studied. Initially, β-CD nanoparticles arrange themselves into linear or channel-like structures, influenced by a complex interplay of favourable and unfavourable interactions with the solvent. These β-CD channels serve as essential structural components that are later assembled into larger spherulitic architectures. The introduction of nanoparticles, including CNCs, MMT, and LAP, is crucial for templating and stabilizing these structures. These nanoparticles enhance inter-channel hydrogen bonding and interfacial alignment, thus facilitating the radial expansion characteristic of spherulitic growth (Fig. 10). This study's findings clearly demonstrate that the structural integrity and viscoelastic properties of β-cyclodextrin-based supramolecular gels can be accurately adjusted by varying both the type and quantity of inorganic fillers. Moderate levels of fillers facilitate the creation of resilient, hierarchically structured networks with high mechanical strength, while excessive filler amounts reduce stiffness but widen the linear viscoelastic region, thereby improving flow capabilities. This presents effective strategies for engineering tunable soft materials.

Additionally, the study confirms that β-cyclodextrin can form stable supramolecular gels through hydrogen bonding and channel-type self-assembly, which can be modified using inorganic fillers like MMT, CNC, or LAP. The findings highlight that careful manipulation of filler content allows for precise control over the gel's mechanical properties, structural organization, and response to shear forces. These insights provide a valuable foundation for developing advanced hybrid gels suitable for applications in controlled release systems, soft actuators, and functional nanocomposites.

## 4. Sensing application

Supramolecular gels are a type of material formed through non-covalent interactions or secondary bonding, which includes hydrogen bonds, van der Waals forces, and π–π interactions. What sets them apart is their ability to respond to different external stimuli, such as changes in temperature, pH levels, exposure to light, or the presence of specific ions or molecules. This adaptability classifies them as smart materials that can alter their properties in response to environmental changes, making them highly valuable for various sensing applications.

### 4.1 Salt sensing

**Fig. 11:**
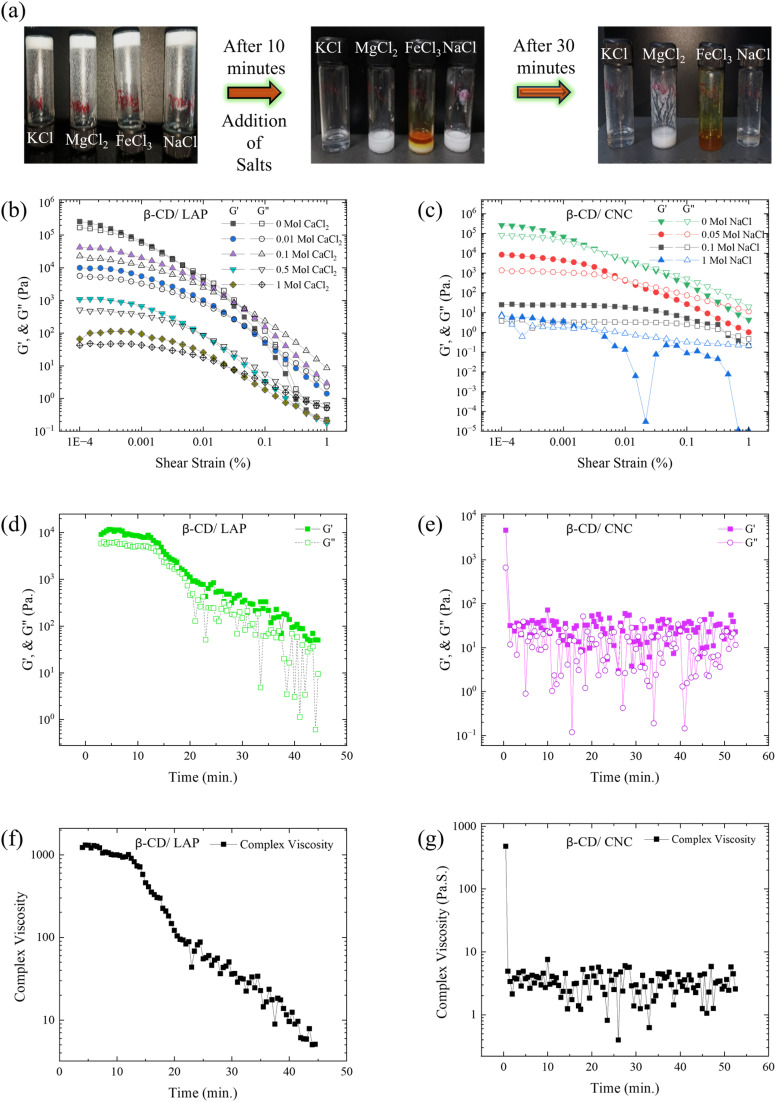
(a) Time-dependent visual response of β-CD gels upon addition of KCl, MgCl_2_, FeCl_3_, and NaCl. The gels remain intact initially, while phase separation, color variation, and eventual collapse over 10–30 min indicate ion-specific disruption of the supramolecular network. (b) Variation of *G*′ and *G*″ for the β-CD/LAP system with increasing CaCl_2_ concentration, showing a sharp decrease in *G*′ and convergence toward *G*″, indicative of network breakdown. (c) Corresponding rheological response of the β-CD/CNC system under NaCl, displaying a more gradual reduction in viscoelastic strength. (d) Time evolution of normalized modulus 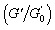, highlighting the higher sensitivity of β-CD/LAP toward 1 M Ca^2+^. (e) Time sweep of β-CD/CNC under 1 M NaCl, showing transition from solid-like (*G*′ > *G*″) to liquid-like (*G*′ ≈ *G*″) behavior. (f) and (g) Complex viscosity *vs.* time for β-CD/LAP (1 M CaCl_2_) and β-CD/CNC (1 M NaCl), respectively, confirming salt-induced network disassembly.

β-Cyclodextrin (β-CD)-based composite gels incorporating nanoparticles are formed through a self-assembly process predominantly driven by intermolecular hydrogen bonding. The resulting three-dimensional network is stabilized by hydrogen bonds among β-CD molecules and between β-CD and the embedded nanofillers, thereby imparting structural integrity to the gel. Upon the introduction of equimolar concentrations of metal salts (NaCl, KCl, MgCl_2_, CaCl_2_, AlCl_3_, and FeCl_3_), pronounced ion-dependent structural changes are observed (Fig. 11a). Although the gels initially remain intact, progressive phase separation, color variation, and eventual collapse over 10–30 min clearly indicate disruption of the supramolecular network.

To substantiate these observations, quantitative rheological measurements were performed (Fig. 11b–g). The β-CD/LAP system exhibits a systematic decrease in storage modulus (*G*′) with increasing CaCl_2_ concentration (0.01–1 M), from ∼10^6^ to 10^2^ Pa, accompanied by convergence of *G*′ and *G*″, indicative of rapid gel–sol transition and network breakdown (Fig. 11b). In contrast, the β-CD/CNC system under NaCl (0.05–1 M) shows a more gradual reduction in viscoelastic strength, with *G*′ decreasing from ∼10^5^ to 10 Pa, reflecting a slower disruption of the network (Fig. 11c).

Time-dependent analysis further reveals a pronounced decay in modulus for the β-CD/LAP system (Fig. 11d), with *G*′ decreasing from ∼10^4^ to 10^1^ Pa in the presence of 1 M Ca^2+^, confirming its higher sensitivity to divalent ions. These measurements were carried out at a constant strain of 0.001% and an angular frequency of 10 rad s^−1^. Consistently, the complex viscosity shows a sharp decline from ∼10^3^ to 5 Pa s for 1 M CaCl_2_ (Fig. 11f). Similarly, the β-CD/CNC system undergoes a progressive transition from solid-like (*G*′ > *G*″) to liquid-like (*G*′ ≈ *G*″) behaviour under 1 M NaCl (Fig. 11g). The corresponding decrease in complex viscosity with time (Fig. 11e and g) further confirms salt-induced network disassembly in both systems. Characteristic collapse times extracted from the rheological profiles provide a direct comparison of ion responsiveness, with the β-CD/CNC system exhibiting faster collapse than the β-CD/LAP system. Overall, these results establish a clear correlation between ion type, supramolecular disruption, and macroscopic gel behaviour, highlighting the tuneable and ion-sensitive nature of β-CD/nanomaterial composite gels for stimuli-responsive applications.

### 4.2 Temperature sensing

**Fig. 12:**
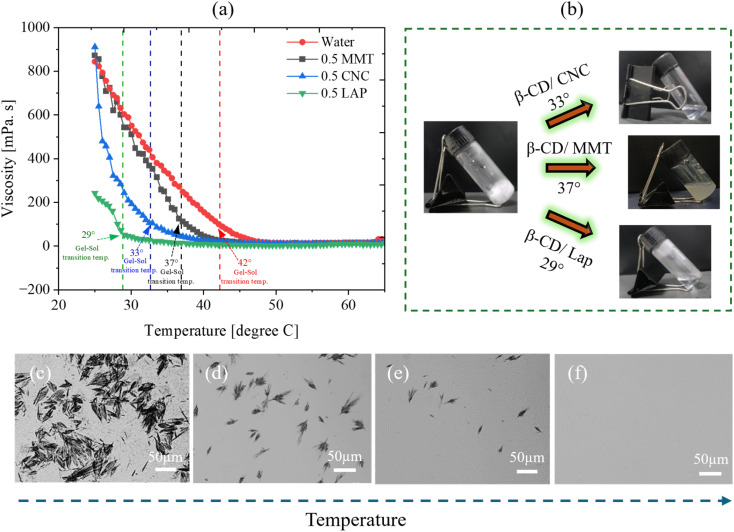
(a) Representation of viscosity *vs.* temperature of β-CD/MMT, β-CD/CNC, and β-CD/LAP systems, (b) appearance of the solution phase while heating for different composites, (c)–(f) POM image for breakdown of gel network while heating from 25° to 35° for β-CD/CNC. (Scale bar 50 µm).

Fig. 12a presents the rheological response during heating, revealing the progressive disruption of the crystalline network over the temperature range of 25–65 °C. The gel–sol transition temperature is strongly dependent on the nature of the nanomaterial incorporated into the β-CD matrix.^51^ Specifically, the critical transition temperatures are 37 °C for β-CD/MMT, 33 °C for β-CD/CNC, and 29 °C for β-CD/LAP. In contrast, the pristine β-CD gel remains stable up to 45.8 °C before undergoing solation, indicating superior thermal stability in the absence of nanofillers.

The reduction in transition temperature upon nanoparticle incorporation can be attributed to the influence of nanofiller geometry and surface chemistry on the supramolecular organization of β-CD. Distinct morphologies—platelets (MMT), rods (CNC), and disks (LAP)—are expected to interact differently with the β-CD lamellae, thereby modulating the network architecture and its associated energy landscape. Consequently, the thermal responsiveness of the gel is systematically altered. Notably, the process is fully reversible, as the system re-forms a gel upon cooling to room temperature (25 °C), as shown in Fig. 12.

Overall, these findings demonstrate that tuning the nanomaterial component enables precise control over the thermal behaviour of the gel. The pronounced temperature sensitivity of the phase transition underscores the potential of β-CD/nanomaterial composite systems as soft, stimuli-responsive materials for temperature sensing applications.

### 4.3 Dye sensing

The removal of dye was quantitatively evaluated using UV-Vis absorption spectroscopy by monitoring the change in absorbance at the characteristic maximum wavelength (*λ*_max_) of the dye. According to the Beer–Lambert law, the absorbance is directly proportional to the dye concentration, allowing absorbance values to be used as a direct measure of concentration during the treatment process.

The percentage of dye removal was calculated using the following equation:
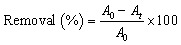
where *A*_0_ is the initial absorbance and *A*_*t*_ is the absorbance at time *t*.

The adsorption performance was further evaluated in terms of adsorption capacity (*q*_*t*_), defined as the amount of dye adsorbed per unit mass of adsorbent at time *t*, calculated as:
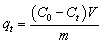
where *C*_0_ and *C*_*t*_ (mg L^−1^) are the initial and time-dependent dye concentrations, respectively, *V*(*L*) is the volume of the dye solution, and *m* (g) is the mass of the adsorbent. At equilibrium, the adsorption capacity is expressed as *q*_e_, obtained by replacing *C*_*t*_ with the equilibrium concentration *C*_e_.

Prior to analysis, all samples were centrifuged or filtered to remove suspended particles, ensuring accurate absorbance measurements. When necessary, samples were diluted to maintain absorbance within the linear range of the instrument. In cases where a calibration curve was established, concentrations were determined more precisely and used for calculating both removal efficiency and adsorption capacity.

**Fig. 13:**
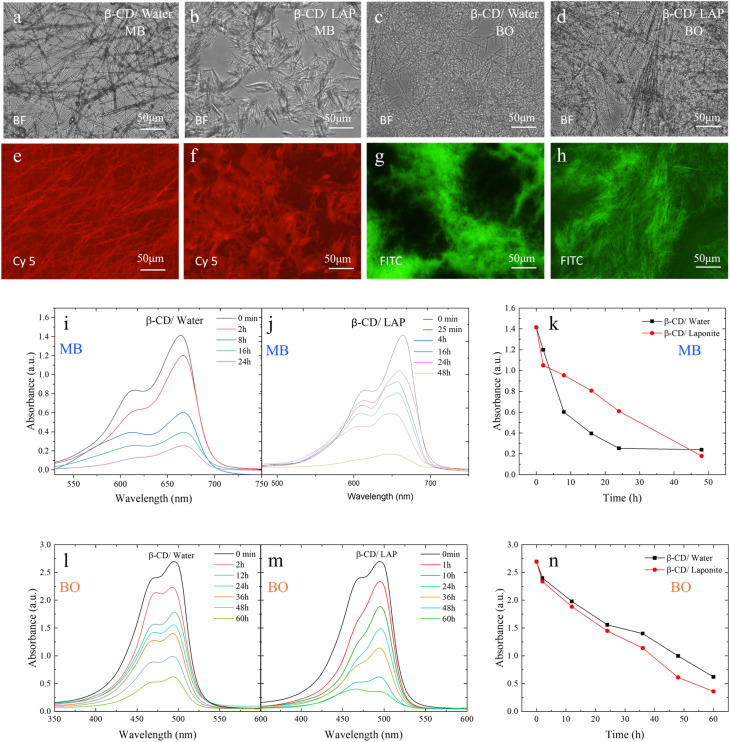
Morphology, fluorescence imaging, and dye sensing behavior of β-CD based systems. (a)–(d) Bright-field (BF) microscopy images showing the microstructure of β-CD in aqueous solution and in the presence of LAP with methylene blue (MB) (b) and (c) and Basic Orange (BO) (a) and (c). Scale bar: 50 µm. (e) and (f) Fluorescence images of MB samples recorded using a Cy5 filter, displaying red emission corresponding to MB distribution in β-CD/water (e) and β-CD/LAP (f) systems. (g) and (h) Fluorescence images of BO samples acquired with a FITC filter, showing green emission in β-CD/water (g) and β-CD/LAP (h) systems. Scale bar: 50 µm. Time-dependent UV-Vis absorption spectra of MB and BO in β-CD/water (i) and (l), and β-CD/LAP (j) and (m) systems at different irradiation times, (k) and (n) time dependent dye removal test for both β-CD/water and β-CD/LAP system for MB and BO dye.

The microstructural features of the β-cyclodextrin (β-CD) systems were first examined by bright-field microscopy (Fig. 13a–d). The β-CD/water system exhibits a network of elongated plate-like structures in the presence of both methylene blue (MB) and Basic Orange 14 (BO), while incorporation of LAPONITE® leads to a denser and more compact morphology, indicative of enhanced supramolecular organization.

Fluorescence imaging further confirms dye incorporation and spatial distribution within the assemblies. MB-containing samples imaged using a Cy5 filter (Fig. 13e and f) display strong red emission, revealing homogeneous dye localization throughout the β-CD matrix, with increased intensity and uniformity in the presence of LAPONITE®. Similarly, BO-loaded systems visualized under a FITC filter (Fig. 13g and h) exhibit bright green fluorescence, with the β-CD/LAP system showing a more continuous and interconnected emission pattern compared to the β-CD/water system. These observations suggest improved dye confinement and distribution within the hybrid system.

The time-dependent dye absorption behavior in both β-CD/water and β-CD/LAP systems was studied using UV-Vis spectroscopy (Fig. 13i–n). The absorbance spectra for various durations, specifically 0 minutes, 30 minutes, 1.5 hours, 4 hours, 16 hours, 24 hours, and 40 hours, were recorded within the wavelength range of 500 to 750 nm for Methylene Blue (MB) (Fig. 13i and j). Over time, the absorbance intensity in the β-CD/water mixture gradually declined, indicating continuous dye removal (Fig. 13i). Notably, the prominent peak of methylene blue, which is located between 660 and 680 nm, showed a significant reduction over the course of 24 hours. This suggests that β-CD effectively interacts with the dye molecules, likely through the formation of inclusion complexes.^56^

The dye removal process appears to consist of two stages: an initial rapid attachment followed by a slower, diffusion-dependent elimination. This is evidenced by a significant decrease in absorbance occurring within the first 1.5 hours, followed by a more gradual decline (Fig. 13d). In contrast, the β-CD/LAP composite system shows a faster and more efficient dye removal mechanism. The reduction in absorbance is more pronounced and occurs more quickly, especially within the first 25 minutes, highlighting the synergistic effect of LAPONITE®. The layered silicate structure of LAPONITE® likely provides additional adsorption sites, enhancing the dispersion of dye molecules. After 40 hours, the absorbance was nearly negligible, indicating that the dye had been almost completely removed. The final dye removal efficiencies were determined to be 83% and 87% for the β-CD/water and β-CD/LAP systems, respectively, based on the decrease in maximum absorbance at *λ*_max_ as shown in Fig. 13k. Under identical conditions (5 mg dye in 100 mL), the β-CD/LAP system exhibits a higher adsorption capacity (8.7 mg g^−1^) compared to β-CD/water (8.3 mg g^−1^), corresponding to removal efficiencies of 87% and 83%, respectively, thereby confirming the enhanced performance arising from the combined effect of host–guest inclusion and LAPONITE®-assisted adsorption.

To prove high absorption behaviour in LAPONITE® based system, test was done with another dye, Basic Orange 14 (BO), characterized by an absorption maximum in the range of 480–500 nm, was used to evaluate the dye removal efficiency of β-CD/water (W) and β-CD/LAP systems. The temporal decrease in absorbance at *λ*_max_ (Fig. 13l–n) serves as a direct indicator of dye removal from solution.

Both systems exhibit a progressive attenuation of the characteristic absorption band with time, confirming continuous dye uptake. However, the β-CD/LAP system demonstrates a significantly enhanced removal performance compared to β-CD/water, highlighting a clear synergistic effect.

Quantitative analysis reveals that the β-CD/water system achieves a removal efficiency of 10.9% at 2 h, increasing to 26.5% at 12 h, 42.2% at 24 h, and 48.0% at 36 h. A more pronounced increase is observed at longer times, reaching 63.0% at 48 h and a maximum removal efficiency of 77.0% after 60 h. In contrast, the β-CD/LAP system exhibits faster kinetics and higher overall efficiency, with removal values of 13.2% at 2 h, 30.1% at 12 h, 46.3% at 24 h, and 57.7% at 36 h. The removal efficiency increases sharply to 77.3% at 48 h and reaches a maximum of 86.6% after 60 h. For an initial dye loading of 9 mg in 100 mL (90 mg L^−1^), the β-CD/LAP system exhibits an adsorption capacity of 15.6 mg g^−1^ at 86.6% removal efficiency, which is significantly higher than that of the β-CD/water system (13.9 mg g^−1^ at 77%), confirming enhanced dye uptake due to the synergistic effect of LAPONITE®.

The findings indicate that β-cyclodextrin (β-CD) can independently enhance the removal of dye molecules through host–guest complexation. However, the addition of LAPONITE® significantly improves both the speed and overall effectiveness of dye removal. This composite of β-CD and LAPONITE® represents a promising hybrid adsorbent for the effective remediation of dyes.

## 5. Conclusions

We have successfully developed innovative hierarchical microstructural gels through the interaction of β-cyclodextrin with small amounts of various negatively charged nanoparticles, such as cellulose nanocrystals, montmorillonite, and LAPONITE®, using a good–poor solvent system. The phase behavior of these gels was significantly influenced by the concentration of nanoparticles and the composition of the solvent, with gelation occurring even at low levels of nanoclay content. Rheological analysis confirmed a decrease in viscoelastic behavior with increasing nanoparticle concentration. These advanced gels exhibit responsiveness to changes in salt concentration and temperature, as well as effective dye removal capabilities, demonstrating their multifunctionality. The temperature-dependent viscosity of these composite systems revealed specific critical temperature thresholds for gel deformation, making them suitable for use as temperature sensors. Additionally, the temperature-induced phase transitions observed across different nanoparticles highlight their significant potential for applications in sensing technologies. The β-CD/nanomaterial composites exhibit enhanced selectivity and sensitivity for detecting cationic dyes, offering a promising foundation for advanced environmental and sensing technologies.

## Conflicts of interest

The authors report no conflicts of interest.

## Supplementary Material

NA-008-D5NA01177A-s001

## Data Availability

The data supporting this article have been included as part of the supplementary information (SI). Supplementary information: particle size (DLS) analysis, optical microscopy of bow-tie microstructures, FTIR spectra, zeta potential measurements, rheological frequency sweep data, XRD analysis, and phase behaviour studies of β-cyclodextrin/nanoparticle composite systems. See DOI: https://doi.org/10.1039/d5na01177a.
