# Supplementary material for: Engineering β-cyclodextrin gels with nanoparticles: tunable assembly and multifunctional applications
Source: Nanoscale Adv. 2026 Apr 30;8(11):3321–39. doi: 10.1039/d5na01177a (PMC13147287; doi:10.1039/d5na01177a)
Supplement: NA-008-D5NA01177A-s001 [file NA-008-D5NA01177A-s001.pdf]

Supplementary Information (SI) for Nanoscale Advances.
This journal is © The Royal Society of Chemistry 2026

Supporting Information

Engineering β-Cyclodextrin Gels with Nanoparticles: Tunable Assembly and Multifunctional Applications

*Sagar Kumar Pathak[1], Aiswarya Sukumaran[1], Ioanna Chazapi[2,3], Claire Hotton[2,†], Erwan Paineau[2], and Ravi Kumar Pujala[1]**

[1]Soft and Active Matter group, Department of Physics, Indian Institute of Science Education and Research (IISER) Tirupati, Andhra Pradesh, 517507, India

[2]Université Paris-Saclay, CNRS, Laboratoire de Physique des Solides, Orsay 91405, France

[3]Université Paris-Saclay, CNRS, CEA, LLB, 91191 Gif-sur-Yvette, France

Corresponding author's E-mail address: pujalaravikumar@labs.iisertirupati.ac.in

Contents	Page no.
1. Particle size: DLS result	2
2. Bow-Tie Microstructures	3-4
3. FTIR of β-CD/ LAP system	4
4. Zeta Potential measurements	5-6
5. Frequency sweep	6-7
6. XRD Analysis	7-8
7. Phase Behaviour	9

1. Particle size (DLS result):

Intensity-weighted hydrodynamic radius (R) distributions obtained from DLS for Laponite (blue), CNC (black), and MMT (red) dispersions. CNC consists of rigid, rod-like particles with monocrystalline domains typically extending 200–400 nm in length, giving intermediate R values. In contrast, Laponite and MMT possess a layered sandwich-like structure with plate-shaped nanoparticles (~1 nm thick) and charged surfaces arising from isomorphic substitution. The Laponite system shows smaller hydrodynamic radii (typically ~50–70 nm), consistent with well-dispersed platelets, whereas the MMT system exhibits larger hydrodynamic radii (typically ~250–350 nm), indicative of tactoidal or aggregated structures. Overall, the hydrodynamic radius follows the trend: Laponite<CNC<MMT

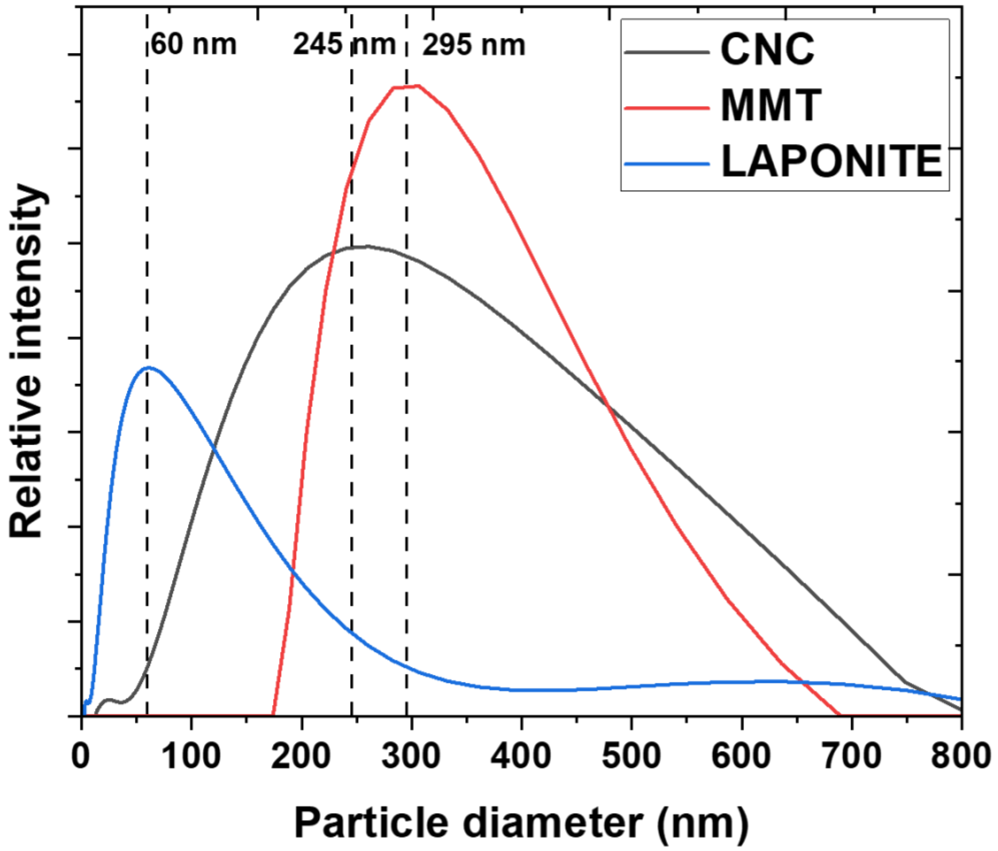

Figure S1. Hydrodynamic radius distribution of anisotropic nanoparticles

2. Formation of Bow-Tie Microstructures in β-Cyclodextrin/Nanomaterial Composites

Microscopic analysis revealed the formation of distinctive bow-tie-shaped microstructures in β-cyclodextrin (β-CD)/nanomaterial composite systems. These unique morphologies emerge as a result of oriented self-assembly and directional crystallization processes mediated by the cooperative interaction between β-CD and layered or rod-like nanomaterials such as Laponite, montmorillonite (MMT), and cellulose nanocrystals (CNC). The bow-tie architecture is characterized by a central narrowed region flanked by two symmetrical, expanded crystalline lobes, resembling the geometry of a bow-tie.

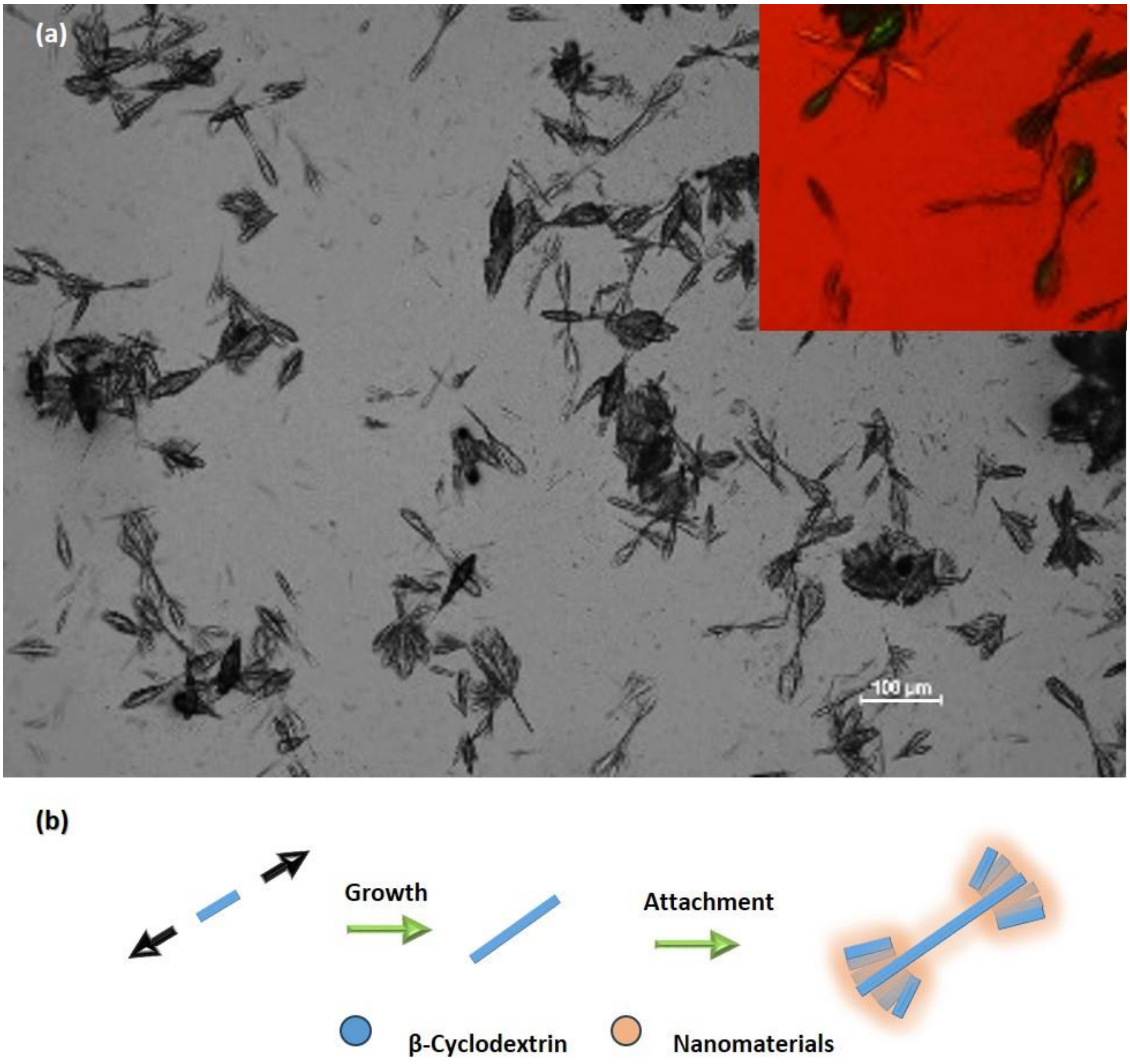

Figure S2: (a) POM image (bright field and cross polariser with lambda plate) of bow-tie microstructures observed for 15 wt% β-CD/ 0.5 CNC during gel formation. (b) Mechanism of formation of bow-tie.

This structure likely originates from the anisotropic growth of β-CD inclusion complexes along specific crystallographic axes, influenced by the nucleating and templating role of the nanomaterial surfaces. The high aspect ratio and surface charge of CNCs and clay platelets facilitate alignment and ordering of β-CD molecules, promoting head-to-tail channel-type packing that expands outward in a two-lobed fashion. The central constriction may result from a localized balance between crystal growth and aggregation suppression due to surface charge repulsion or concentration gradients during solvent evaporation or supersaturation.

Such morphology is not typically observed in β-CD alone, suggesting that the presence of the nanomaterials alters the self-assembly pathway, introducing symmetry-breaking growth modes that give rise to these complex supramolecular geometries. The bow-tie structure provides a promising platform for host–guest encapsulation, directional molecular diffusion, or optical anisotropy, and may serve as a novel design element in the development of functional materials with controlled morphology and internal architecture.

3. FTIR of β-CD/ LAP system:

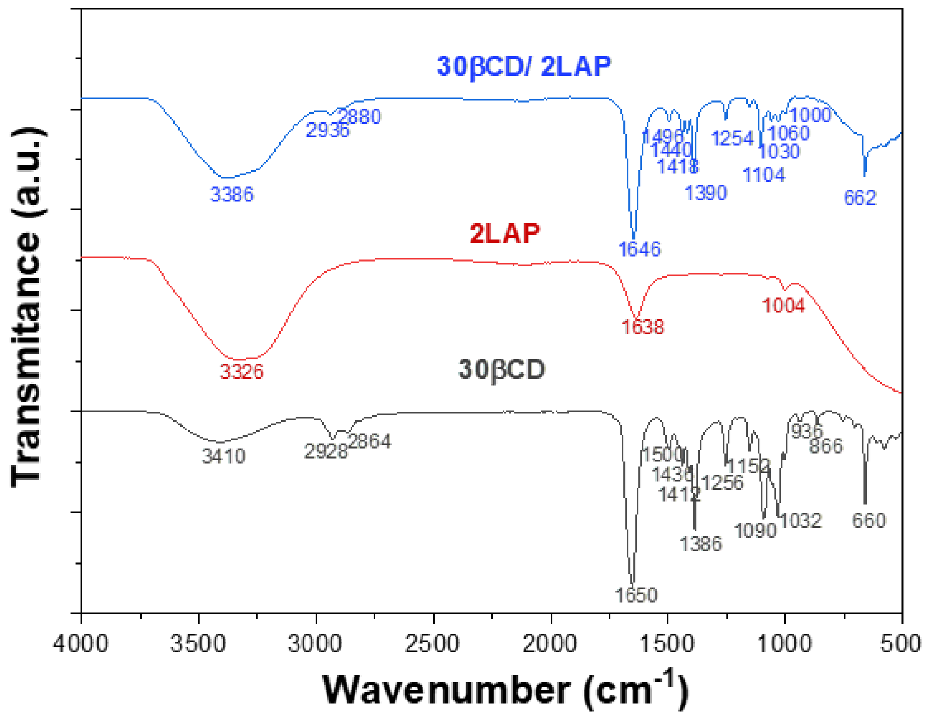

Figure S3: represents the FTIR plot, for β-CD/ Laponite system.

4. Zeta Potential Behaviour in β-Cyclodextrin/Nanomaterial Composites

The zeta potential behavior of β-cyclodextrin (β-CD)-based composite systems was investigated upon increasing the concentration of various negatively charged nanomaterials—cellulose nanocrystals (CNC), montmorillonite (MMT), and Laponite—from 0.5 wt% to 2 wt%. Interestingly, in all three composite systems, the zeta potential shifted from strongly negative to moderately negative, indicating a progressive reduction in surface charge magnitude. Specifically, in the β-CD/CNC system, the zeta potential increased from −44 mV to −8 mV; for the β-CD/MMT system, from −30 mV to −2 mV; and for the β-CD/LAP system, from −28 mV to −1 mV as the concentration of the respective nanomaterial was raised.

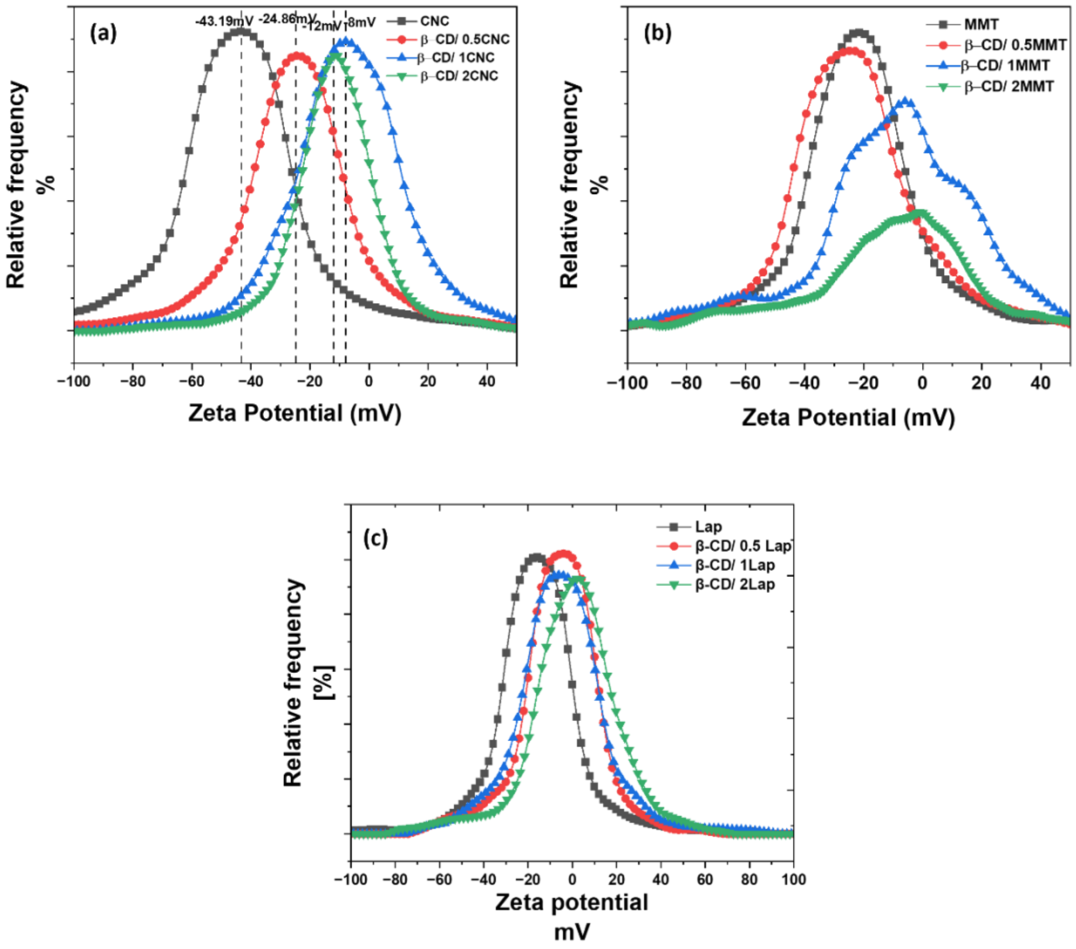

Figure S4: (a), (b), and (c) represent the zeta potential measurements for three different composite systems β-CD/CNC, β-CD/MMT, and β-CD/LAP, respectively, with increasing nanomaterial concentrations.

This counterintuitive trend—where the surface potential becomes less negative with increasing nanomaterial content—suggests that β-CD interacts closely with the anionic surfaces of CNC, MMT, and Laponite, potentially shielding or partially neutralizing the surface charges. As more nanomaterial is added, a denser network of β-CD/nanomaterial interactions likely forms, resulting in surface-adhered or intercalated structures that limit the free mobility of negative functional groups, such as sulfate esters (in CNC) or silanol moieties (in MMT and Laponite). The adsorption or complexation of β-CD molecules may mask these charges, thereby reducing the overall zeta potential.

Additionally, increased solid content could lead to the formation of more aggregated or compact supramolecular structures, in which electrical double layers are compressed, and zeta potential measurements reflect an apparent reduction in net surface charge. The magnitude of zeta potential reduction followed the order: CNC > MMT > Laponite, consistent with differences in their surface chemistries and specific interactions with β-CD.

Overall, the observed decrease in surface charge magnitude with increasing nanomaterial concentration highlights the complex interplay among electrostatic, hydrogen-bonding, and inclusion interactions within these hybrid assemblies. This tunability in surface potential is critical for controlling colloidal stability, phase behavior, and potential host–guest applications in supramolecular nanocomposites.

5. Frequency sweep

Rheological characterization provided further insight into the structural dynamics of the β-CD/Nanoparticles systems. Frequency sweep tests (Figure S5) verified a predominantly elastic response (G' > G") across all frequencies, although increased MMT content was observed to lessen the gel's overall stiffness. Furthermore, flow curve data (Figure 4d) illustrated shear-thinning behavior characteristic of non-Newtonian fluids. For CNC-based assemblies, frequency sweeps revealed that the loss modulus (G″) exceeded the storage modulus (G′) at higher concentrations, signifying a predominantly viscous response and the inability of CNC to form a stable elastic network at elevated loadings (Figure S5b). This shift toward liquid-like behavior is indicative of weakened supramolecular interactions and a reduction in long-range structural integrity.

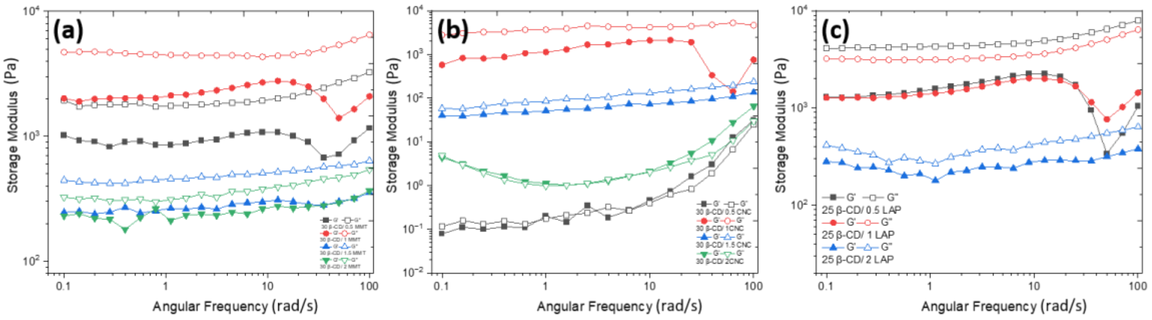

Figure S5: Rheological behaviour of β-CD/nanoparticle systems. (a) Frequency sweeps of MMT composites showing viscous-dominated behavior (G″ > G′) at higher concentrations. (b) Frequency sweeps of β-CD/CNC composites with reduced G′ and liquid-like response at higher Laponite loadings. (c) Frequency sweeps of β-CD/ Laponite composites.

A comparable trend was observed for the β-CD/Laponite composites. With increasing Laponite concentration (0.5–2 wt%), the storage modulus decreased markedly from ~10^6 Pa to ~10^4 Pa (Figure S5c). This reduction in elasticity suggests that instead of reinforcing the gel network, excess Laponite interferes with the organization of β-CD channel-type assemblies, leading to a partial disintegration of the supramolecular framework. Consequently, the system displays a viscoelastic liquid character, as evidenced by G″ dominating over G′ across the entire frequency spectrum.

6. XRD in β-Cyclodextrin/Nanomaterial Composites

X-ray diffraction (XRD) analysis was used to investigate the structural changes in the layered nanomaterials upon incorporation of β-cyclodextrin (β-CD). As shown in Fig. S1 and Table S1, the (001) diffraction peaks corresponding to the basal spacing of Laponite (Lap), cellulose nanocrystals (CNC), and montmorillonite (MMT) exhibited a noticeable shift toward lower angles with increasing β-CD content. This shift corresponds to an increase in d-spacing from 13.87 Å to 14.11 Å for Laponite, 13.57 Å to 14.03 Å for CNC, and 13.80 Å to 14.12 Å for MMT, indicating a consistent trend across all systems.

The observed expansion in interlayer distance is attributed to the intercalation of β-CD molecules into the interlayer galleries of the 2D clay-based nanosheets. The inclusion of β-CD likely occurs via non-covalent interactions, such as hydrogen bonding and van der Waals forces, between the hydroxyl groups of β-CD and the surface functionalities of the nanomaterials. This structural modification suggests a successful integration of β-CD into the

layered host matrices and supports the formation of stable hybrid assemblies with modified supramolecular architectures

Sample	2θ	d- Spacing (Å)
MMT	7.37	11.97
LAP	6.7	13.16
CNC	14.8	5.97
β-CD	6.25	14.12
30β-CD/ 0.5 MMT	6.29	14.03
10β-CD/ 2 MMT	6.51	13.57
30β-CD/ 0.5CNC	6.26	14.11
10β-CD/ 2 CNC	6.4	13.80
30β-CD/ 0.5 LAP	6.26	14.11
10β-CD/ 2 LAP	6.37	13.87

Table S1: XRD d-spacing values for different ratios of CD and nanoparticles

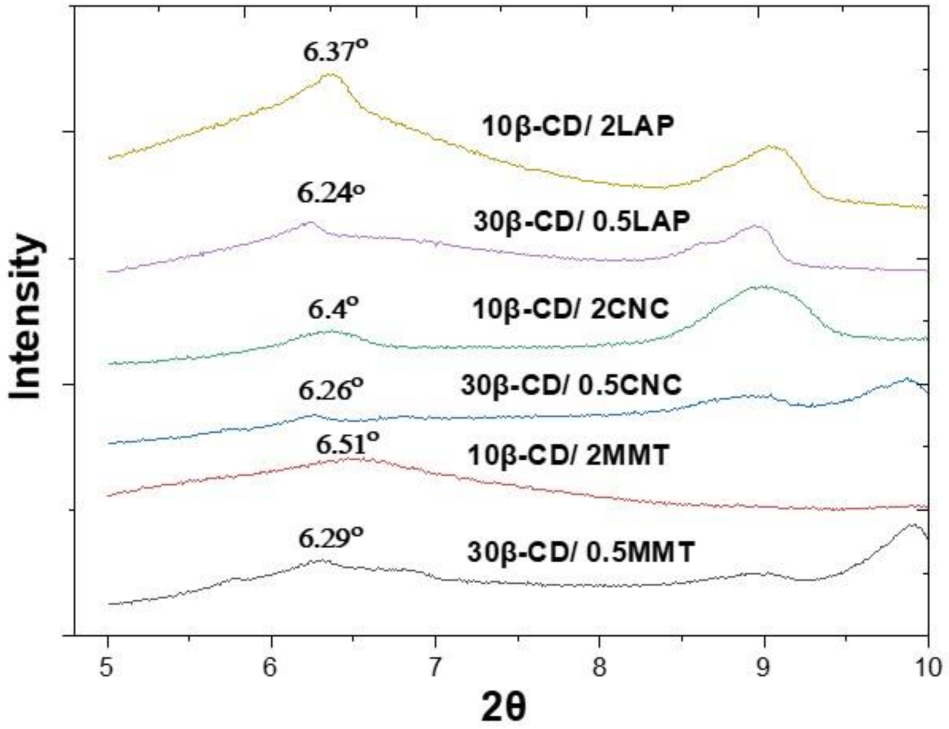

Figure S6: XRD d-spacing values for different ratios of CD and nanoparticles

7. Phase Behaviour:

β-CD(DMF) / Water		β-CD(DMF) / 0.5- 1 MMT		β-CD(DMF) / 1.5- 2 MMT	
Starting	Ending	Starting	Ending	Starting	Ending
1:4	7:3	1:4	6:1	1:6	4:1
β-CD(DMF) / Water		β-CD(DMF) / 0.5- 1 CNC		β-CD(DMF) / 1.5- 2 CNC	
Starting	Ending	Starting	Ending	Starting	Ending
1:4	7:3	3:4	6:1	1:1	4:1
β-CD(DMF) / Water		β-CD(DMF) / 0.5 LAP		β-CD(DMF) / 1- 2 LAP	
Starting	Ending	Starting	Ending	Starting	Ending
1:4	7:3	1:4	6:1	1:6	6:1

Table S2: Comparison table of phase behavior for β-CD/ Water, β-CD/ MMT, β-CD/ CNC, and β-CD/ Laponite systems.

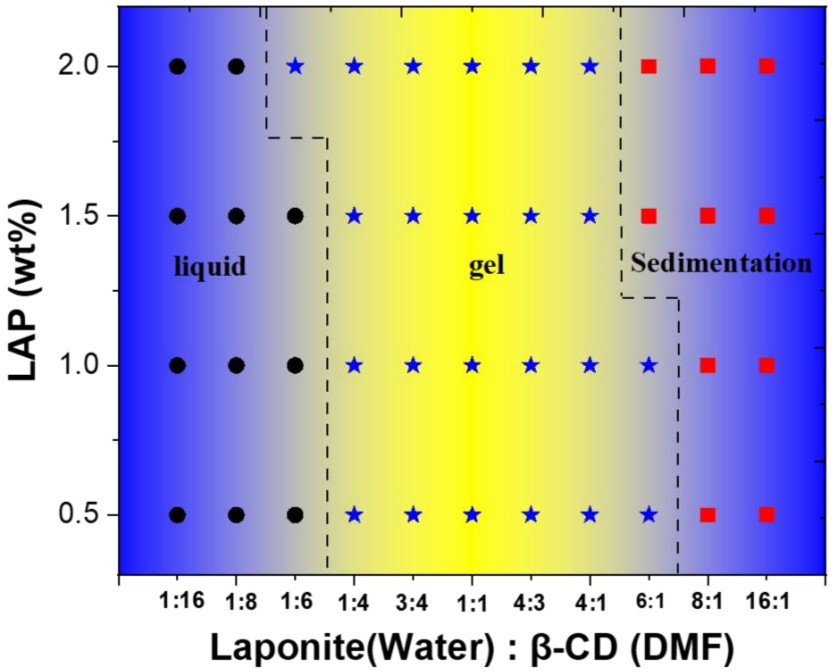

Figure S7: represented the phase behaviour of β-CD/ Laponite system, which is similar to β-CD/ MMT system.
